# Anxiety, depression, and regret of donation in living kidney donors

**DOI:** 10.1186/s12882-018-1024-0

**Published:** 2018-09-04

**Authors:** Courtenay M. Holscher, Joseph Leanza, Alvin G. Thomas, Madeleine M. Waldram, Christine E. Haugen, Kyle R. Jackson, Sunjae Bae, Allan B. Massie, Dorry L. Segev

**Affiliations:** 1 Department of Surgery, Johns Hopkins University School of Medicine, 2000 E. Monument St., Baltimore, MD 21205 USA; 2 Department of Epidemiology, Johns Hopkins School of Public Health, Baltimore, MD USA

**Keywords:** Kidney transplantation, Living donors, Donor follow-up, Quality of life, Anxiety, Depression, Mental health screening, Psychiatric screening, GAD-2, PHQ-2

## Abstract

**Background:**

Previous studies have reported a wide range of prevalence of post-donation anxiety, depression, and regret in living kidney donors (LKDs). It is also unclear what risk factors are associated with these outcomes.

**Methods:**

We screened 825 LKDs for anxiety and depression using 2-item GAD-2 and PHQ-2 scales and asked about regret.

**Results:**

Overall, 5.5% screened positive for anxiety, 4.2% for depression, and 2.1% reported regretting their donation. While there was moderate correlation between positive anxiety and depression screens (*r* = 0.52), there was no correlation between regret and positive screens (*r* < 0.1 for both). A positive anxiety screen was more likely in LKDs with a positive depression screen (adjusted relative risk [aRR] 13.72, 95% confidence interval [CI] 6.78–27.74, *p* < 0.001). Similarly, a positive depression screen was more likely in LKDs with a positive anxiety screen (aRR 19.50, 95% CI 6.94–54.81, *p* < 0.001), as well as in those whose recipients experienced graft loss (aRR 5.38, 95% CI 1.29–22.32, *p* = 0.02). Regret was more likely in LKDs with a positive anxiety screen (aRR 5.68, 95% CI 1.20–26.90, *p* = 0.03). This was a single center cross-sectional study which may limit generalizability and examination of causal effects. Also, due to the low prevalence of adverse psychosocial outcomes, we may lack power to detect some associations between donor characteristics and anxiety, depression, or regret.

**Conclusions:**

Although there is a low prevalence of anxiety, depression, and regret of donation among LKDs, these are interrelated conditions and a positive screen for one condition should prompt evaluation for other conditions.

## Background

Anxiety and mood disorders affect 18.1 and 9.5% of US adults per year, and have been associated with higher rates of disability, non-compliance, acute illness, exacerbations of chronic illness, and even death [1–6]. Although pre-donation psychiatric assessments for all living kidney donors (LKDs) are mandated in the United States by the Organ Procurement and Transplantation Network (OPTN) [7]. LKDs still experience adverse psychosocial outcomes post-donation [8–11]. Not only is this a bad situation for the donor: when LKDs experience adverse psychosocial outcomes, the transplant community is also at risk of losing advocates for living kidney donation. However, our understanding of post-donation anxiety, depression, and regretting the donation remains poorly understood.

First, the post-donation prevalence of these conditions remains unclear, with wide ranges reported of 6–67% for anxiety, 5–23% for depression, and 0–7% for regretting the donation [9, 12–21]. Beyond unclear prevalence estimates, our understanding is limited by a paucity of studies identifying risk factors for these conditions. In a cohort of 45 Portuguese donors, 67% had post-donation anxiety; however, risk factors for anxiety were not studied [19]. Wiedebusch et al. studied 161 German LKDs and found a 21% prevalence of anxiety and that both anxiety and depression were associated with worse quality of life [21]. A higher risk of depression has been reported in unmarried LKDs, those with a history of pre-donation depression, greater financial burden, recipient graft failure or death, and short-term medical complications or re-hospitalizations [16, 18, 19, 22]. Though prior studies have reported that most LKDs would willingly donate again [8–10, 12, 13, 21, 23–34], it is intuitive that those donors with negative psychosocial outcomes post-donation might be at higher risk for regretting their donation.

To better understand prevalence and risk factors associated with anxiety, depression, and regret in LKDs, we screened a cohort of LKDs using the 2-item Generalized Anxiety Disorder scale (GAD-2), the 2-item Patient Health Questionnaire (PHQ-2) scale for depression, and a single-question survey on regret of donation.

## Methods

### Study population

The Wellness and Health Outcomes in Live Donors (WHOLE-Donor) cohort is an ongoing study of living kidney donors who underwent donor nephrectomy at Johns Hopkins Hospital between 1982 and 2015, for which enrollment began in 2011. Participants consented to quality of life surveys and a survey detailing medical, surgical, hospitalization, psychiatric, and social history. This is a cross-sectional study of participants’ first survey responses at the time of enrollment from 2011 to 2017. The Johns Hopkins Medicine Institutional Review Board (NA_00044282) approved this study.

### Outcomes

We defined post-donation anxiety as a score of 3 or higher on the GAD-2 anxiety screen, as previously defined and validated [35]. We defined post-donation depression as a score of 3 or higher on the PHQ-2 depression screen, as previously defined and validated [36, 37]. Post-donation regret was evaluated using the question, “Given the chance, would you offer to donate your kidney again?” We defined post-donation regret as those who gave negative responses on the 5-point Likert scale, i.e. “probably not” or “definitely not.”

### Risk factors

Demographic characteristics and medical history including comorbid diagnoses and medications were self-reported by participants. Antidepressants, anti-anxiety medications, medications for obsessive-compulsive disorder, and medications for bipolar disorder were considered psychiatric medications. Difficulty changing or obtaining new health insurance or life insurance were self-reported. A socioeconomic status (SES) index corresponding to each participant’s ZIP code was generated using the method described by the Agency for Healthcare Research and Quality, based on crowding, property value, poverty levels, educational attainment, employment, and median household income at the ZIP code level [38]. Participants were classified as having below median SES or above median SES within our study population based on the ZIP code they reported. A random subset of 204 participants also completed a survey module which included questions about relationship with recipient and recipient graft and vital status.

### Model selection

We used modified Poisson regression models to describe the relative risk of covariates for each of the outcomes [39]. In order to select the best subset of covariates, we compared models including different subsets of covariates using the Akaike information criterion, an indicator of the relative quality of models. The covariates were age, number of years since donation, gender, African American race, college education or higher, married or living with a partner, employment status, current or former tobacco smoking, socioeconomic status, hypertension, diabetes, chronic kidney disease, hyperlipidemia, diagnosis of depression, diagnosis of another psychiatric illness, development of another comorbidity, and development of any comorbidity. Additionally, positive GAD-2 screen, positive PHQ-2 screen, and reporting regret of donation were included in each subset of covariates except for the model where that risk factor was considered the outcome.

Because 204 participants were randomized to the model containing questions about recipient outcomes, we initially excluded these 204 from covariate selection described above, and created a model offset to constrain covariate coefficients to the values determined by the initial regression models. In order then to include recipient outcomes as risk factors for anxiety, depression, and regret of donation, we used the model offset from the prior step with robust standard errors to run Poisson regression models. By using this method to constrain coefficients, we were able to maintain power but also study the risk factors assessed in a survey module administered to a subset of our study population.

### Statistical analysis

Descriptive statistics are reported using t tests, chi-square tests, and Pearson’s correlation coefficient. An α of 0.05 was considered significant. Confidence intervals are reported as per the method of Louis and Zeger [40]. Analyses were performed using Stata 14.2/SE for Windows (College Station, Texas).

## Results

### Study population

Among 825 participants, median (interquartile range [IQR]) time since donation was 6 (3-10) years, median (IQR) age at survey completion was 46 (36–54) years, 63.5% of participants were female, 10.1% were African American, 66.7% had some college education or higher, 70.9% were married or living with their partner, and 63.2% were employed full-time (Table 1). Overall 47.0% had developed one or more comorbid illnesses including high cholesterol (19.8%), hypertension (15.2%), diabetes (3.6%), chronic kidney disease (1.7%), depression (13.3%), other psychiatric disorders (5.3%), and other diseases (39.6%) including thyroid disorders and hematologic disorders. There were 436 participants who responded to questions about insurance (question response rate 52.8%); of them, 44 (10.1%) reported difficulty obtaining or changing life or health insurance (Table 1).

**Table 1:** Characteristics of living kidney donors

	All	Anxiety	Depression	Regret
	*n* = 825	*n* = 41	*n* = 31	*n* = 16
Age at survey completion, median years (IQR)	46 (36–54)	44 (36–51)	44 (38–55)	39 (34–55)
Time since donation, median years (IQR)	6 (3–10)	6 (3–8)	6 (3–11)	7 (5–10)
BMI at survey completion, median (IQR)	26 (24–30)	26 (24–30)	26 (23–29)	28 (25–31)
Female	524 (63.5%)	26 (63.4%)	21 (67.7%)	12 (75.0%)
African American	83 (10.1%)	4 (9.8%)	4 (12.9%)	5 (31.2%)*
Some college education or higher	550 (66.7%)	21 (51.2%)*	11 (35.5%)*	10 (62.5%)
Married or living with partner	583 (70.9%)	21 (51.2%)*	14 (45.2%)*	7 (43.8%)*
Current or former smoker	309 (37.4%)	24 (58.5%)*	18 (58.1%)*	8 (50.0%)
Low SES by ZIP code of residence	360 (50.1%)	21 (61.8%)	17 (63.0%)	5 (50.0%)
Employment status				
Full-time	521 (63.2%)	27 (65.8%)	18 (58.1%)	9 (56.2%)
Part-time	94 (11.4%)	7 (17.1%)	3 (9.7%)	1 (6.2%)
Retired	129 (15.6%)	3 (7.3%)	5 (16.1%)	4 (25.0%)
Unemployed	81 (9.8%)	4 (9.8%)	5 (16.1%)	2 (12.5%)
Any comorbidity	388 (47.0%)	23 (56.1%)	20 (64.5%)	11 (68.8%)
Hypertension	125 (15.2%)	11 (26.8%)*	12 (38.7%)*	3 (18.8%)
Diabetes	29 (3.6%)	1 (2.6%)	2 (6.7%)	1 (6.2%)
Chronic kidney disease	14 (1.7%)	0	0	0
High cholesterol	163 (19.8%)	9 (22.0%)	8 (25.8%)	6 (37.5%)
Diagnosis of depression	103 (13.3%)	9 (26.5%)	10 (35.7%)*	5 (31.2%)
Diagnosis of other psychiatric disorder	44 (5.3%)	4 (9.8%)	3 (9.7%)	2 (12.5%)
Other comorbidity	327 (39.6%)	18 (43.9%)	16 (51.6%)	9 (56.2%)
Any psychiatric medication use	135 (16.4%)	14 (34.2%)*	9 (29.0%)*	3 (18.8%)
Trouble obtaining or changing insurance^a^	44 (10.1%)	2 (7.7%)	1 (5.3%)	3 (30.0%)*
Relationship to recipient^b^				
Child	25 (12.2%)	3 (25.0%)	3 (23.1%)	1 (20.0%)
Parent	17 (8.3%)	0	0	0
Sibling	43 (21.1%)	0	1 (7.7%)	2 (40.0%)
Spouse/partner	41 (20.1%)	2 (16.7%)	1 (7.7%)	1 (20.0%)
Friend	29 (14.2%)	4 (33.3%)	3 (23.1%)	0
Other	49 (24.0%)	3 (25.0%)	5 (38.5%)	1 (20.0%)
Recipient graft loss^b^	20 (14.1%)	0	2 (28.6%)	1 (50.0%)
Recipient mortality^b^	61 (29.9%)	5 (41.7%)	6 (46.2%)	3 (60.0%)

*p < 0.05 compared to subjects without anxiety, depression, or regret, respectively

^a^A total of 436 subjects responded to a question about experience with life or health insurance

^b^A total of 204 subjects participated in the survey module which included questions about recipients

Of the 204 participants who participated in the survey module that included questions about their recipient, 21.1% reported donating to their sibling, 20.1% reported donating to a spouse or partner, 14.2% reported donating to a friend, and 12.2% reported donating to a child; 29.9% reported that their recipient had since died and 14.1% reported that their recipient had experienced graft loss.

### Prevalence and correlation of anxiety, depression, and regret

Forty one (5.5%) of 742 respondents screened positive for anxiety (89.9% response rate). Respondents to the anxiety screen were similar to non-respondents in age, race, education, employment status, and marital status, but were less likely female (62.4% vs. 73.5%, *p* = 0.046). 31 (4.2%) of 741 respondents screened positive for depression (89.8% response rate). Respondents to the depression screen were similar to non-respondents in age, gender, race, education, employment status, and marital status. 16 (2.1%) of 749 respondents reported feeling regret of their donation (90.8% response rate). Respondents to the question about regret were similar to non-respondents in age, gender, race, education, employment status, and marital status. While there was moderate correlation between a positive anxiety screen and a positive depression screen (*r* = 0.52), there was no correlation between regret and anxiety or depression (*r* < 0.1 for both, Table 2).

**Table 2:** Correlation between anxiety, depression, and regret following living kidney donation

	Anxiety	Depression	Regret
Anxiety	1.00		
Depression	0.52	1.00	
Regret	0.05	0.02	1.00

### Risk factors for anxiety

Compared to subjects who did not screen positive on the GAD-2, subjects with a positive GAD-2 screen were less likely to have some college education or higher (51.2% vs. 67.5%, *p* = 0.03), less likely to be married or living with a partner (51.2% vs. 72.6%, *p* = 0.003), more likely to be a current or former smoker (58.5% vs. 37.5%, *p* = 0.007), and more likely to have hypertension (26.8% vs. 15.3%, *p* < 0.001, Table 1). In multivariable analysis, subjects with a positive PHQ-2 depression screen were more likely to have a positive GAD-2 anxiety screen (adjusted relative risk [aRR] _6.78_ 13.72 _27.74_, *p* < 0.001), while those who donated more recently were less likely to have a positive GAD-2 (per year, aRR _0.89_ 0.93 _0.98_, *p* = 0.006, Table 3).

**Table 3:** Risk factors associated with positive GAD-2 anxiety screen in living kidney donors**.** Adjusted relative risk presented with 95% confidence interval. Model adjusted for risk factors included in the table

	Adjusted Relative Risk	*p*-value
Positive PHQ-2 screen	_6.78_ 13.72 _27.74_	< 0.001
Years since donation (by year)	_0.89_ 0.93 _0.98_	0.006
Married/living with a partner	_0.26_ 0.52 _1.05_	0.07
Hypertension	_0.96_ 1.54 _2.48_	0.08
Recipient alive	_0.38_ 0.82 _1.78_	0.6

### Risk factors for depression

Compared to subjects who did not screen positive on the PHQ-2, subjects with a positive PHQ-2 screen were less likely to have some college education or higher (35.5% vs. 68.0%, *p* < 0.001), less likely to be married or living with a partner (45.2% vs. 72.2%, *p* = 0.001), more likely to be a current or former smoker (58.1% vs. 37.5%, *p* = 0.02), more likely to have hypertension (38.7% vs. 14.8%, *p* < 0.001), and more likely to report having been diagnosed with depression by a doctor or other healthcare professional (35.7% vs. 12.2%, *p* = 0.004, Table 1). In multivariable analysis, subjects with a positive GAD-2 anxiety screen (aRR _6.94_ 19.50 _54.81_, *p* < 0.001) and those whose recipient had experience graft loss (aRR _1.29_ 5.38 _22.32_, *p* = 0.02) were more likely to have a positive PHQ-2 depression screen (Table 4).

**Table 4:** Risk factors associated with positive PHQ-2 depression screen in living kidney donors. Adjusted relative risk presented with 95% confidence interval. Model adjusted for risk factors included in the table

	Adjusted Relative Risk	*p*-value
Positive GAD-2 screen	_6.94_ 19.50 _54.81_	< 0.001
Some college education or higher	_0.18_ 0.50 _1.36_	0.2
Married/living with a partner	_0.24_ 0.68 _1.88_	0.4
Hypertension	_0.90_ 1.59 _2.79_	0.1
Diagnosis of depression	_0.48_ 1.45 _4.40_	0.5
Recipient graft loss	_1.29_ 5.38 _22.32_	0.02

### Risk factors for regret

Compared to subjects who did not report regret, subjects who reported regretting their donation were more likely to be African American (31.2% vs. 9.7%, *p* = 0.005), less likely to be married or living with a partner (43.8% vs. 72.0%, *p* = 0.01), and more likely to report having trouble obtaining or changing health or life insurance (30.0% vs. 10.2%, *p* = 0.04, Table 1). In multivariable analysis, subjects with a positive GAD-2 anxiety screen were more likely to regret donation (aRR _1.20_ 5.68 _26.90_, *p* = 0.03, Table 5).

**Table 5:** Risk factors associated with regret of donation in living kidney donors. Adjusted relative risk presented with 95% confidence interval. Model adjusted for risk factors included in the table

	Adjusted Relative Risk	*p*-value
African American	_0.75_ 3.78 _18.92_	0.1
Age at survey completion (per 10 years)	_0.58_ 0.98 _1.65_	0.9
Positive GAD-2 screen	_1.20_ 5.68 _26.90_	0.03
Development of any comorbidity	_0.35_ 1.53 _6.74_	0.6
Trouble obtaining or changing insurance	_0.75_ 3.13 _12.98_	0.1
Recipient graft loss	_0.57_ 4.59 _36.81_	0.2

## Discussion

In this cohort of 825 LKDs interviewed at a median of 6 years after donation, 5.5% screened positive for anxiety, 4.2% screened positive for depression, and 2.1% reported regretting their donation. We found moderate correlation between positive anxiety and depression screens (*r* = 0.52). A positive anxiety screen was associated with a nearly 20-fold higher risk for positive depression screen and a 5.7-fold higher risk for regret of donation. Similarly, a positive depression screen was associated with a 13.7-fold higher risk for positive anxiety screen. On univariate analysis, college education, marital status, smoking history, and diagnosis of hypertension were associated with anxiety and depression, while African American race, marital status, and insurance problems were associated with regret.

Our finding that 5.5% of LKDs screen positive for anxiety is similar to the 6% of a cohort of 48 Australian donors [9], lower than the 21% with anxiety in a cohort of 161 German LKDs [21], and substantially lower than the 67% with anxiety in a cohort of 45 Portuguese LKDs [19]. The prevalence of anxiety in LKDs is also substantially lower than a US general population prevalence estimate of 18.1% [1]. Similar to findings from the RELIVE study, development of comorbid illness, namely hypertension, was associated with both depression and anxiety in our cohort [16].

Our finding of an association between marital status and anxiety, depression, and regret is consistent with a Japanese study which reported that living alone was the single best predictor for depression in kidney recipients [41]. The prevalence of depression in this cohort of 116 Japanese donors was 41.4%, however, much higher than the 4.2% prevalence in our cohort. Interestingly, though only 4.2% screened positive for depressive symptoms, 13.3% reported a history of depression. This is consistent with prior community studies which have found that half of those with a first episode of major depression recover within 3 months [42, 43] and that half had no future episodes of major depression [43]. Our finding that age was not associated with anxiety or depression must be considered in the context of conflicting literature. Isotani et al. report better quality of life scores for younger donors, while other groups have reported that younger donors have worse quality of life in a variety of domains [12, 16, 18, 26, 29].

Our finding that 2.1% of LKDs would not donate again is within the range of reported prevalence of regret, ranging from 0 to 7% [8, 12, 13, 21, 23–32, 44]. Given the increased emphasis on holistic donor follow-up and support for wellbeing, a benefit of addressing mental health might lead fewer LKDs to regret their donation, as we found a positive anxiety screen was associated with regret.

Our study does have several limitations. The study was conducted in a single center, which may limit the generalizability of our findings. However, the age, sex, and race of our participants is similar to the general population of US donors [45]. Also, despite a sample size of 825 donors and excellent response rate, we may lack power to detect additional associations between donor characteristics and anxiety, depression, or regret of donation in multivariable regression, due to the low prevalence of these outcomes. Additionally, our study used screening tools to describe symptoms of anxiety and depression rather than a diagnostic tool; however, by using a screening tool we were able to capture these symptoms with good sensitivity using the GAD-2 and PHQ-2. Finally, although the cross-sectional nature of our survey prohibits examination of causal effects, the prevalence estimates and risk factors provide insight in LKD follow-up and might help identify those LKDs most in need of formal referral for mental health services.

## Conclusions

In this cohort of 825 LKDs, we found that 5.5% screened positive for anxiety, 4.2% screened positive for depression, and 2.1% reported regret of their decision to donate. Given the growing waitlist and an emphasis on expanding live donation, the finding that some LKDs harbor regret is not only ethically relevant, but also a practical consideration in pre-donation counseling. While the OPTN does not specifically mandate psychosocial follow-up post-donation, they emphasize consideration of donor safety and wellbeing. Our study suggests that psychosocial screening at follow-up may prove especially useful for patients with other psychosocial symptoms, who develop a chronic medical condition after donating, or who lack a proximal social support network.

## Abbreviations

aRR: Adjusted relative risk
BMI: Body mass index
CI: Confidence interval
GAD-2: Generalized Anxiety Disorder
IQR: Interquartile range
LKD: Living kidney donor
OPTN: Organ Procurement and Transplantation Network
PHQ-2: Patient Health Questionnaire
SES: Socioeconomic status
WHOLE: Wellness and Health Outcomes in LivE Donors
